# Comment on “Can Charge Transfer Across C─H···O Hydrogen Bonds Stabilize Oil Droplets in Water?”

**DOI:** 10.1002/anie.8842163

**Published:** 2026-05-27

**Authors:** P. Singh, C. E. Rani, S. Pullanchery, S. Roke

**Affiliations:** ^1^ Laboratory for Fundamental BioPhotonics (LBP) Institute of Bio‐engineering (IBI), École Polytechnique Fédérale de Lausanne (EPFL) Lausanne Switzerland; ^2^ Department of Chemistry College Station Texas A&M University Texas USA; ^3^ Institute of Materials Science (IMX) School of Engineering (STI), École Polytechnique Fédérale de Lausanne (EPFL) Lausanne Switzerland; ^4^ Lausanne Centre for Ultrafast Science (LACUS) École Polytechnique Fédérale de Lausanne (EPFL) Lausanne Switzerland

## Abstract

Kinetically stable oil nanodroplets in water display an electrophoretic mobility linked to a negative surface charge. The source of this charge is proposed to arise from the adsorption of hydroxide ions or through charge transfer (CT), that is displacement of electronic charge across improper C─H···O hydrogen bonds. Zhao et al. [R. Q. Zhao, H. Y. Shen, R. A. Lacour, J. P. Heindel, M. Head‐ Gordon, and T. Head‐Gordon, Angewandte Chemie‐ International Edition 64 (2025): e202508145, https://doi.org/10.1002/anie.202508145.] questioned the possibility of CT across an oil droplet interface based on density functional theory simulations of hexane molecules in water. Here, we introduce the two prevalent explanations for the surface charge on oil droplets in water, hydroxide adsorption and CT through improper H‐bonds, showing that, while the hydroxide hypothesis is most intuitive, there is no molecular level experimental evidence from a range of surface specific techniques. We correct a misconception about the origin of the blue shift in the original sum frequency scattering work [S. Pullanchery, S. Kulik, B. Rehl, A. Hassanali, and S. Roke, Science 374 (2021): 13661370, https://doi.org/10.1126/science.abj3007.], and comment on the choice of hexane clusters in water: Hexane does not form kinetically stable droplets in water, as it is 9 orders of magnitude more soluble in water than hexadecane. Without exhibiting kinetic stability—the behavior the simulations seek to explain—there is limited predictive ability on CT through improper H‐bonds as a mechanism to stabilize droplets.

## Graphical abstract

Nanoscale oil droplets in water are kinetically stable and charged, with a pH dependent electrophoretic mobility. Although the source of this charge is agreed to arise from water, 2 main hypotheses remain: OH^−^ adsorption or electronic charge density displaced via hydrogen bonds. Molecular surface specific experiments are reviewed, that all point to the charge transfer hypothesis. Dismissing it based on a simulation of a cluster of hexane in water is inappropriate as hexane does not form kinetically stable droplets in water.

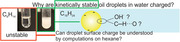

## 1 Introduction

The mixing of water and oil depends on the chain length of the oil molecules. Hexane has a solubility of 110 mM and hexadecane has a solubility of 93 pM [1] in water. Continuous interfacial films are formed when short‐ and long‐range interactions favor wetting, which happens for very short (< C_4_) alkanes. Wetting is not observed when both short‐ and long‐range interactions disfavor wetting (> C_8_). In an intermediate regime (C_5_‐C_8_) where the short‐range interactions favor wetting, but the long‐range interactions do not favor wetting, partial wetting is observed [2, 3]. Nevertheless, when ultrapure hexadecane (C_16_) is mixed with ultrapure water and ultrasonicated, kinetically stable oil droplets in water are formed with sizes in the 100–300 nm range. Hexadecane droplets are kinetically stable for several days [1, 4] and display a negative electrophoretic mobility that is pH dependent, increasing by ∼x 2–3 when the pH is changed from neutral to 11 [5]. The overarching question, which is also the theme of the paper by Zhao et al. [6], is: Why are these droplets kinetically stable?

This question has been asked for many years and has received increased attention recently. Because the droplets display a negative electrophoretic mobility, it is concluded that there is some form of charge on them. Electrophoretic mobilities are in the range −1.5 to −5.5 × 10^−8^ m^2^/Vs, which converts to ζ‐potential in the |30 – 120| mV range [5], (albeit under assumptions that are potentially fragile, as discussed later on). Taking these values as surface potentials and applying the Poisson‐Boltzmann equation [7], this corresponds to surface charge densities in the range ∼ 0.01 (100) – 0.31 (3.32) e^−^/nm^2^ (nm^2^/e^−^). There are three main types of explanations for the presence of charge:

1. **Hydroxide adsorption**. Hydroxide, the negatively charged self‐ion of water, offers perhaps the most intuitive explanation for the sign of mobility and its pH dependence [8, 9]: Under pH neutral condition OH^−^ ions are already adsorbed at the interface, and the adsorbed amount increases by x 2–3 when the bulk pH is increased to 10–11 (0.1‐1 mM). Increased surface charge leads to increased droplet mobility. This is the oldest and most widely cited (and questioned [10]) hypothesis for the source of charge on oil droplets in water.
2. **Improper H‐bonds with charge transfer from water to oil**. This explanation emerged in the 2010s [11] in response to negative results in molecular level experimental investigations and theories concerning the surface structure of the aqueous interface and the specific‐ion nature of hydroxide. In this scenario, improper hydrogen (H)‐bonds between water and oil [12] (H‐O···H‐C) are the source of interfacial charge. H bonds consist of several interactions, one of which is CT. Such H‐bonds are called “weak” or “improper” because the charge that is transferred to the oil is much smaller than in regular H‐bonds resulting in a contraction and a blue shift of the C‐H stretch mode of the accepting bond [13].
3. **Impurities**: Several studies have brought forth the possibility that impurities are responsible for the pH dependence in the ζ‐potential. Theoretical works have hinted at pH dependent dissociation in combination with ionic strength induced surface density enhancement [14]. Experimental works have hypothesized that impurities arise from the oil, in the form of carboxylates [15]; the water (in the form of an undefined agent with a specific pK_a_ [16], or atmospheric CO_2_ [17]); or desorption from the walls of the glassware when it is subjected to >24 h of sonication [4, 18]. Since Zhao et al. [6] do not consider this mechanism and focus on water‐originating explanations, we mention it when discussing data that excludes impurity as a possibility and here refer to earlier in‐depth considerations published in Refs [4, 19, 20].

Here, we comment on the choice of hexane as prototypical oil in the simulations by Zhao et al. [6]., who used it to conclude that “CT is negligible across oil‐droplet‐water interfaces”. We first summarize key surface and molecular specific experimental results from the past ∼25 years and discuss them in relation to the two hypotheses that derive from the properties of water itself. This is followed by comments on the work by Zhao et al. [6].

## 2 Results and Discussion

### 2.1 Hypothesis 1: Hydroxide

#### 2.1.1 Detecting OH^−^ Ions at Planar and Droplet Interfaces

During the late 20^th^ century, the molecular level structure of aqueous interfaces could be studied in detail, due to the development of X‐ray photoelectron spectroscopy, resonant and off‐resonant second harmonic generation (SHG), and vibrational sum frequency generation (SFG), applied to planar or droplet interfaces [21]. This allowed for the direct detection of specific molecules such as OH^−^. Since the estimated adsorbed surface charge densities at pH 10–11 is ∼0.31 e^−^/nm^2^ (3.2 nm^2^/e^−^) [9], it should be possible to detect it on the surface with surface‐specific spectroscopies. OH^−^ has specific energy levels that can be detected: the O 2p‐π valence electron [22], a charge‐transfer‐to‐solvent transition at 187 nm [23], and the O‐H^−^ (O‐D^−^) vibrational stretch mode at ∼3620 (2700) cm^−1^ in Raman [24, 25], and ∼3600 (2685) cm^−1^ in IR spectroscopy [24, 26]). Surface tension measurements on the planar aqueous‐air interface [27], and on the hexane‐water interface [28] have established that the surface tension is practically invariant in the pH range 1–13 [27] and increases at pH > 14. The presence of OH^−^ at the air‐water interface was probed with resonant SHG in reflection mode [29]. Hydroxide ions could be detected at the interface above bulk concentrations of 5 M, (pH > 14). X‐ray photoelectron spectroscopy [30] showed the presence of interfacial and sub‐surface hydroxide ions at concentrations >1 m. Reflection mode SFG experiments [31] showed very little difference between the neat air‐water interface and the interface between air and a 1.2 M NaOH solution in water. The explicit detection of hydroxide at the surface at concentrations >1 M was explained with the aid of specific ion effect theories and MD simulations [32], reflecting ideas from the early 20^th^ century [10]: Ions that lower the surface tension and show some partition to the interface are generally “soft”, large and polarizable (such as I^−^ and SCN^−^, which lower the interfacial tension), while ions that do not partition to the interface are “hard”, small, and not polarizable (such as F^−^, which increase the interfacial tension) [10, 32]. Hydroxide, being small and having favorable interactions with water prefers to reside in the bulk solution, leading to the well‐known trend in the change in surface tension [33] (Δγ) that Δγ(SCN^−^)<Δγ(I^−^)<Δγ(Br^−^)<Δγ(Cl^−^)<Δγ(F^−^)<Δγ(OH^−^). For all ions investigated, surface adsorption starts to appear ∼ >0.5 M, which would be in a pH range 13–14, not pH 9, 10 or 11 (0.1 – 1 mM). This data is summarized in Table 1.

**TABLE 1:** **Key experimental observations** regarding the presence of hydroxide ions at equilibrated planar interfaces and kinetically stable hexadecane nanodroplet water interfaces.

Probe	Interface	Specificity to OH^−^	Detected?
Surface tension	Planar air‐water [27] Planar oil‐water [28]	No (interfacial energy) No	γ ↑ > 1 M γ ↑ > 1 M
XPS	Planar air‐water [24]	O 2pπ valence electron	>1 m
Resonant reflection SHG	Planar air‐water [29]	charge‐transfer‐to‐solvent (187 nm)	>5 M
Non‐resonant reflection SHG	Planar hexadecane‐water interface [34]	No	Changes in the SH intensity vs. pH, caused by interference effects from changes in ionic strength [35]
Reflection SFG	Planar air‐water [31] Planar PDMS‐water [36] Planar OTS‐silica‐water [37] Planar hexane‐water [38]	O‐H^−^ (3600‐3620 cm^−1^) / O‐D^−^ stretch vibration (2685–2700 cm^−1^)	No change >3600 cm^−1^ measured up to 1.2 M, [31] 1 M, [36] pH 11, [37] pH 10 [38]
SFS	C_16_ droplets in aqueous solution [11, 39, 40]	O‐H^−^ (3600‐3620 cm^−1^) / O‐D^−^ stretch vibration (2685–2700 cm^−1^)	Entire spectral range pH independent (up to pH 12.5)
Non‐resonant SHS	C_16_ droplets in aqueous solution [11, 39, 40]	No (electrostatic field at interface)	Intensity and scattering patterns pH independent

#### 2.1.2 Kinetically Stable Oil Nanodroplets

**FIGURE 1:**
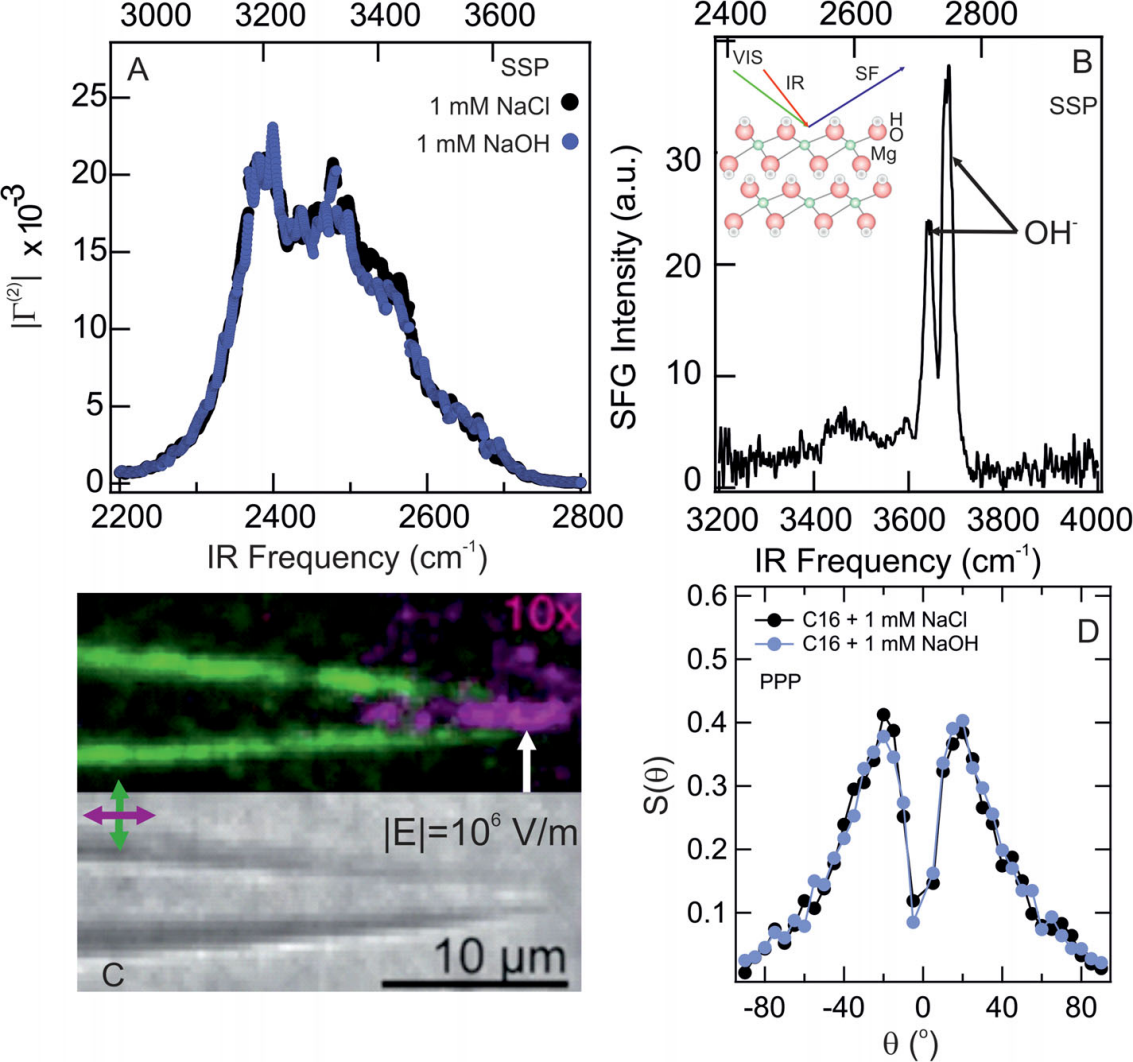
Molecular level probing of hydroxide and surface electric fields. (A) Vibrational SFS spectra of O‐D stretch modes of 2 vol% hexadecane droplets in D_2_O at pD 7 (1 mM NaCl, black) and pD 11 (1 mM NaOD, blue) recorded using the SSP polarization combination. Data adapted from Ref. [40]. (B) Reflection SFG spectrum of a Mg(OH)_2_ surface in ambient atmosphere, recorded in the SSP polarization combination. (C) Bottom: Phase contrast image of a microcapillary in 10 mM NaCl aqueous solution. Top: SH images, recorded in orthogonal polarization directions as indicated by the colored arrows, of the micropipette in the same solution but now subject to an electric bias applied by two electrodes, one placed inside the capillary and the other outside, close to the 1 micron opening. The electrodes are not visible in the image. At an external bias of −10 V the residual field inside the capillary opening is 10^6^ V/m which gives rise to significant water alignment that is responsible for the (purple) SH contrast. Figure adapted from Ref [43]. (D) Angle‐resolved SHS patterns, of 0.05 vol% hexadecane droplets in 1 mM NaCl (black data) and 1 mM NaOH (blue data) measured in the PPP polarization combinations. There is no influence of pH on the electrostatic surface potential and the electric double layer. Data adapted from Ref [40].

Equilibrated planar air‐water and oil‐water interfaces cannot be investigated in terms of their electrophoretic properties and may not have identical structures to kinetically stabilize oil nanodroplet interfaces that are made through homogenization or ultrasonication. To make a better connection to the observations of electrophoretic mobility on kinetically stable oil droplets in water, hexadecane droplets at neutral pH and pH 11 were investigated using both electrophoretic mobility measurements as well as surface specific vibrational sum frequency scattering (SFS). In these experiments ∼1 vol% nano‐emulsions having ∼120 nm averaged droplet radii displayed increased mobility with pH (x 2.2 from pH 7 to 11 at constant ionic strength) [40], in agreement with earlier experimental data [5, 8]. The interfacial C─H and O─D stretch modes were recorded using different polarization combinations [39, 40, 41, 42]. The O─D stretch SFS spectra for different pH values are shown in Figure 1A. The O─D stretch spectra [40, 41] consist of a single broad feature that cannot be unambiguously fitted into separate modes. Instead, combining different spectra measured at different polarization combinations with computed nonlinear optical responses of O─D stretch modes having different symmetries, a broad non‐H‐bonded water feature with C_∞v_ symmetry was identified at 2550–2750 cm^−1^, significantly (red‐)shifted to lower frequencies, compared to the free O‐D C_∞v_ stretch mode at the air‐water interface. This spectral region would also be the one where the stretch mode of the hydroxide ion should be found (at 2680–2700 cm^−1^). However, there is no such peak. The absence of this vibrational mode in the SFS spectrum at both pH values suggests that the interfacial number density of hydroxide ions at the interface is below the detection limit (∼0.036 e^−^/nm^2^; 27 nm^2^/e^−^ [19]).

#### 2.1.3 Reflection SFG versus SFS from Nanodroplets

Reflection mode SFG spectra from the planar hexane‐water interface [38] display pH dependent changes in the intensity of the water O‐H stretch band, and the SFG spectra of the C‐H region that reports on the hexane conformation also changes. Both features – the broad water band, and the hexane C‐H stretches – do not change in the SF scattering spectra as a function of pH on hexadecane oil nanodroplets (in multiple polarization combinations). This difference suggests that both experiments probe different structural effects. The ionic strength in the solution is kept constant in the droplet experiments, but not in the planar interface experiments and this is known to have a big effect on the SFG intensity of the water band [35, 44]. Further differences in conclusions arise from interpretation: The reflection mode hexane‐water experiments are interpreted as follows [38]: Assuming that (i) the intensity of the water band at 3200–3600 cm^−1^ reports on the surface potential which relates to the surface charge via the Gouy‐Chapman model, and that (ii) this surface charge is originating from OH^−^ adsorption, a saturation coverage of 1.55 nm^2^/OH^−^ is derived. Accompanying MD simulations propose a layer of oriented hydroxides at the interface. With such high concentrations, it is puzzling why there is no indication of hydroxide ions in the vibrational SFG spectrum. The SFS spectra were interpreted to arise from charge transfer through improper H‐bonds, which is an explanation that does not invoke the need for adsorbed ions as charge carriers, as there is no evidence for them in the data (see below for more details).

Besides the differences in interpretation, though, it is important to point out that the planar hexane‐water interface [38] does not show any pH dependent spectral changes above 3590 cm^−1^, which agrees with the droplet experiments in Figure 1A. In both cases OH^−^ is not distinctly detected at the aqueous interface, which agrees with the experiments summarized in Table 1.

#### 2.1.4 Is Hydroxide Invisible to Vibrational Surface Spectroscopy?

The absence of a specific hydroxide vibrational mode from the SFG spectra of the planar and droplet surfaces can alternatively occur if interfacial hydroxides are invisible to SFG, for example, because their cross‐section for an SFG transition is too weak or because they are oriented in such a way that they are invisible. To investigate this possibility, we performed (i) reflection SFG experiments on a surface that contains hydroxide, and (ii) second harmonic scattering (SHS) on hexadecane droplets in water. SHS reports on surface charge as it samples the residual interfacial electric field. If changing the pH adds surface charges, even if these are randomly oriented, they should change the SHS patterns/intensity.

Figure 1B shows reflection SFG spectra obtained from the surface of a Mg(OH)_2_ crystal. A sketch of the structure of Mg(OH)_2_ is provided in the inset of Figure 1B. The interfacial OH^−^ groups have two OH^−^ stretch modes, one symmetric (A_1g_) and one antisymmetric (A_2u_). The OH^−^ stretch modes of Mg(OH)_2_ are also both IR and Raman active [45], meeting the selection criterion for detecting the topmost molecular layer(s) of OH^−^ with SFG. Figure 1B shows the SFG spectrum using the SSP polarization combination, showing two distinct peaks (3640 cm^−1^, assigned to A_1g_ and 3680 cm^−1^, assigned to A_2u_) that correspond to the stretch modes of the crystal interface. The broad spectral feature at lower frequencies is arising from O─H stretch modes of adsorbed interfacial water as the experiment was performed under ambient conditions. Thus, Figure 1B shows that if OH^−^(OD^−^) is present on the surface, it can be detected by its specific vibrational modes in an SFG experiment. In aqueous solutions the OH^−^(OD^−^) stretch mode is a single mode in the region 3600 – 3620 cm^−1^ / 2680 – 2720 cm^−1^.

Even when the cross‐section points to SF‐detectable hydroxide ions, they could still be SF‐invisible if the nonlinear polarization was oscillating in an orientation parallel to the interface, in which case the mode would be suppressed in SFG. This would, however, still lead to additional surface charge. To determine the change in surface charge as a function of pH, the hexadecane nanodroplets were investigated with non‐resonant angle‐resolved second harmonic scattering (AR‐SHS) [35, 46]. Non‐resonant SHS can be used to extract the surface potential (Φ_0_), which is defined as the integral from infinity in the bulk up to the surface plane of the materials’ residual electric field in the medium. This residual electric field is the sum of all the electrostatic fields in the medium [47]. This is different from the localized “reaction field” at a single molecular group that is reported on by Stark field spectroscopy [48]. The surface potential Φ_0_ is also different from the ζ‐potential, which is a phenomenological entity derived from electrophoretic mobility under the assumption that this mobility is originating from the movement of adsorbed ionic species [7]. SHG has been used in the form of Electric Field Induced SHG (EFISH [49]) since the 1960's and can be used to measure electric fields in media [49, 50]. As an example, Figure 1C shows a 250 ms acquisition SH image from Ref [43] of a borosilicate glass capillary (top, white light image) with a 1‐micron wide opening. Inside and outside the capillary electrodes are positioned with an electric bias, such that the field inside the 1‐micron opening is 10^6^ V/m (= 10^−2^ MV/cm). The contrast in the SH image, measured by orthogonal polarization combinations, reports on oriented water in the capillary‐aqueous interface (green, with an interfacial probing thickness <1 nm), and water oriented by the external field (purple). This shows that SHG is very sensitive to electric fields and electric double layer structure.

Two independent angular resolved SHS scattering patterns recorded for different polarization combinations can be measured from the nanodroplet solutions. Fits to solutions to Maxwell's equations provide values for the one remaining second‐order surface susceptibility element and the surface potential. Changes in the surface charge density, ionic strength and electric field adjacent to the interface lead to distinct changes in the scattering patterns [51]. Figure 1D shows SHS patterns recorded in the PPP polarization ratio for solutions, having a bulk concentration of 1 mM NaCl (pH 7) and 1 mM NaOH (pH 11) (from Ref [40].). These SHG data might appear to disagree with reflection‐mode SHG experiments on planar hexadecane‐water surfaces by Gan et al. [34] which showed a maximum at pH 11 (1 mM). This was interpreted as surface‐accumulation and saturation of hydroxide ions. However, the maximum in the SHG response is a common, long unexplained feature, that arises from ionic strength‐dependent variations in probing depth [35]. At ∼1 mM the number of water molecules that contribute to the response is maximized which is why there is a maximum in intensity. For SHS and SFS, such effects lead to different scattering patterns which can be exploited to retrieve better accuracy in extracting relevant parameters [51].

The SHS patterns in Figure 1D were theoretically reproduced with a surface potential (Φ_0_) of Φ_0_ = −32 mV. The global error bar in this estimation (±25 mV) arises from uncertainty in literature values of known constants used to compute the surface potential from the experimental data [46]. The patterns are not changing with pH under conditions of constant ionic strength [40].

That this data is invariant under pH, both in terms of intensity and in terms of shape means that, upon increasing the pH:

- No increased adsorption of charged species occurs, which excludes parallel or randomly orientated hydroxide as well as the adsorption of anions involved in impurity‐based explanations.
- No changes in the bulk ionic strength.
- Interfacial residual electric fields do not exceed ∼10^7^ V/m (∼0.1 MV/cm).

### 2.2 Improper H‐bonds and Charge Transfer

As summarized in Table 1, various label‐free surface specific methods aimed to probe OH^−^ specifically (through CTTS transitions, 2p‐π transitions or O‐H^−^ stretch modes) do not provide any evidence for a pH dependent interfacial accumulation of hydroxide below pH ∼13 – 14 (100 mM – 1 M), either at the planar aqueous interfaces or at hexadecane droplet‐in‐water interfaces. Direct measurements of the interfacial potential with AR‐SHS show no evidence of any change in charge distribution. Therefore, an alternative explanation is required; preferably, one that explains the following phenomena:

- Hydrophobic nanoparticles and droplets of hexadecane and other materials all display the same pH‐dependent change in electrophoretic mobility [5, 8, 9, 52, 53, 54, 55]: Increasing the bulk pH to a mildly basic value (∼11) results in a x 2–3 increase in mobility under the influence of an external electrostatic field.
- The same pH dependent behavior is seen in other electrophoretic phenomena: pressure‐driven electro‐osmosis through hexagonal boron nitride nanocapillaries [56], carbon nanotubes [57], graphene oxide membrane conductance [58], and streaming currents through MoS_2_ pores [59] all show the same amplification as the mobility.

All these substrates display vastly different surface chemistries in aqueous solutions, which is unlikely to lead to the same behaviour. Therefore, an explanation is required that originates from the water itself, being the only common component in all these experiments. The explanation should not involve the adsorption of ionic species as it would not agree with the surface spectroscopy experiments discussed above.

In the charge‐transfer‐through‐improper‐H‐bonds explanation, improper hydrogen (H)‐bonds between water and oil (H─O···H─C) are the source of interfacial charge. Such H‐bonds have been proposed based on NMR, vibrational spectroscopy and x‐ray diffraction experiments [12, 60]. H bonding, a highly cooperative phenomenon exists of several interactions, including CT and Pauli repulsion. The charge that is transferred to the C─H groups of the oil is much smaller than in regular H‐bonds, resulting in a contraction and a blue shift of the C─H stretch mode of the donating bond [13]. The contraction and consequential blue shift are due to Pauli repulsion, being now relatively more important for the balance of interactions than in ordinary (O─H···O) H‐bonds in which CT is much larger (which results in a red shift). This blue shift has been observed in the C─H modes of hexadecane droplets and it correlates with the sign of the mobility and the existence of water‐oil contacts: the blue shift vanishes on a deliberately positively charged droplet or one that has been “protected” by a full monolayer of other molecules [41].

We explained this blue shift earlier by: (p. 4, Ref [41].), “The blue shift in C─H modes arises from C─H bond contraction (28, 29). In H bonds in water, charge transfer is the primary contributor to all interactions, leading to bond lengthening and a red shift. C─H···O bonds have relatively weak charge transfer, and thus the Pauli repulsion between the filled C─H and O orbitals dominates the interactions (29), resulting in a blue shift (for details, see the Supporting Information, section S7). The charge transferred from water to oil was in the range of 0.025 to 0.05 electrons / dodecane molecule (12). Although the energetic stabilization of single C─H···O H bonds is rather weak (a fraction of the thermal energy), the collective contribution of such small interactions can lead to a sufficient buildup of charge on the oil droplet, as predicted by Poli et al. (12).” Zhao et al. [6] write: “The lack of charge transfer also explains why vibrational sum frequency scattering reports a blue shift in the oil C─H frequency when forming emulsions with water, which arises from Pauli repulsion due to localized confinement at the interface.” thereby implying there is a contradiction with our work, but on this particular point our explanations are, in fact, identical.

This mechanism potentially provides a net negative charge which is needed to stabilize the droplets, without invoking the direct adsorption of ions, which is required in the hydroxide or impurity hypotheses, but not observed in any of the discussed label‐free interface specific experiments (Table 1).

**FIGURE 2:**
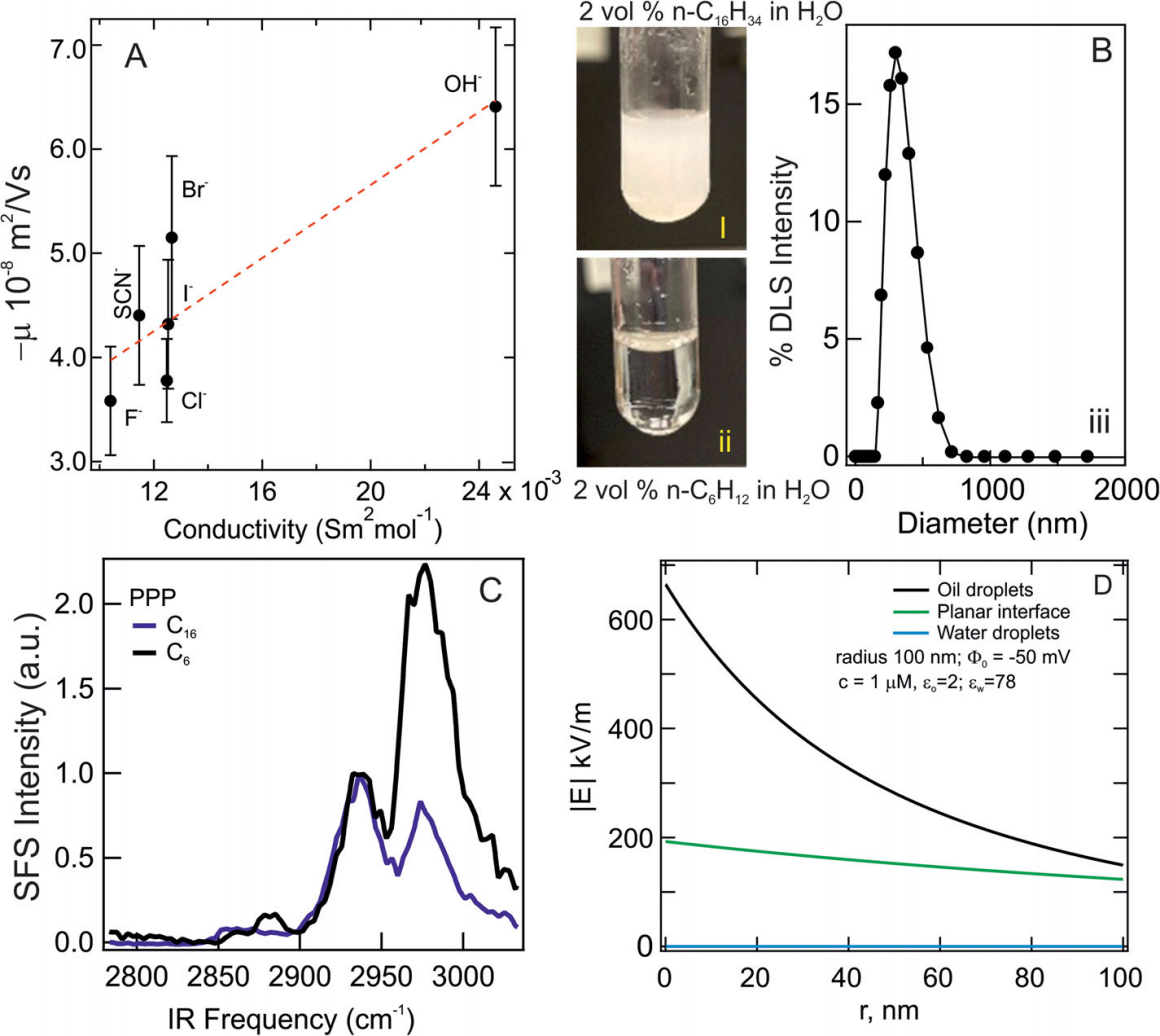
Droplet properties and the effect of chain length of the oil. (A) Mobility of hexadecane droplets in aqueous solutions vs. their conductivity, the aqueous solution is prepared with different salts at a concentration of 0.3 mM. (B) Nano‐emulsification of hexane versus hexadecane in water. Photos taken of the resultant sample directly after emulsifying 2 vol% hexadecane (n‐C_16_H_34_, Sigma Aldrich, analytical standard), (i), and hexane (Sigma‐Aldrich, analytical standard), (ii) in ultrapure water. A typical droplet size distribution obtained from dynamic light scattering for hexadecane nanodroplets in water (iii). This could not be measured for hexane, as the resultant sample consisted of phase‐separate liquids as seen immediately after retrieval from the ultrasonic bath. (C) SFS response in the C‐H region of hexane vs hexadecane droplets (1 vol %, R∼95 nm) having a dilute surface coverage of deuterated dodecyl sulfate (>4.25 nm^2^/DS^−^). (D) Magnitude of the electrostatic field that penetrates the water phase outside a 100 nm oil droplet in water, away from a planar extended oil interface in contact with water, and towards the inside of water droplet in oil. Computations were made by solving the Poisson‐Boltzmann equation, which has different solutions arising from different boundary conditions in these 3 different cases (see Ref.i [61]), leading to different interface and macroscopic properties. Constants used in the computation: droplet radius: 100 nm, surface potential Φ_0_ = −50 mV, ionic strength, c = 1 µM, dielectric constant of oil, ε_o_ = 2, and water ε_w_ = 78.

The pH‐dependent increase in mobility is commonly attributed to higher droplet velocity arising from increased adsorption of ionic charge and the resulting electrostatic force. However, in the absence of molecular‐level evidence for ionic adsorption, the same effect may originate from reduced resistance of the aqueous phase to charge motion, that is, increased liquid conductance. Conductance measurements record the electric current through a medium under an applied bias, whereas mobility measurements determine the velocity of charged objects via light scattering under a similar bias. Both probe charge transport but are not specific to the nature of the charge. Displacement fields in liquids are motions of charge, which can arise from ions, but also from electronic charges, or propagating defects as long as these eventually lead to a detectable current. Consistently, aqueous conductivity and droplet mobility show the same pH dependence: increasing the pH from 7 to 11 enhances both the conductivity and the mobility [5, 8, 9, 52, 53, 54, 55] by a factor of 2.2. Likewise, the electrophoretic phenomena of Refs [56, 57, 58, 59]. change by the same amount, suggesting the behaviors are not a surface but a bulk effect. Figure 2A shows a correlation between mobility and conductivity obtained for different electrolyte solutions. Note that the same experiment cannot be performed on acidic solutions as the droplets are unstable under acidic conditions.

### 2.3 Comments on the Exclusion of the Improper H‐Bond‐Charge Transfer Mechanism

Zhao et al. [6]. concluded that “CT is negligible across oil‐droplet‐water interfaces”, from a computation on hexane molecules in water. The computations showed a spread in values, dependent on the precise geometry of the water‐hexane contacts. Adding forward and backward CT, a net transfer of 0.0007 e^−^/nm^2^ remained, which was argued to be insufficient to produce the amount of charge needed to stabilize the hexadecane droplets. The CT found by Zhao et al., was ∼ 20 x smaller than that computed by Poli et al., who computed CT across a 4.6 x 4.6 x 6.2 nm^3^ dodecane/water slab. This poses several questions / comments:

- Is hexane the relevant prototypical oil molecule to determine the nature of the charge on kinetically stable oil droplets in water?
- Is a cluster of a few hexane molecules in water sufficient to capture electronic polarization effects near an oil‐water interface or an oil droplet?
- What is the actual surface charge distribution?

Below we consider these questions.

#### 2.3.1 Is Hexane the Relevant Prototypical Oil Molecule to Answer the Question About What Charges Oil Droplets in Water?

If hexane is the correct choice to represent the expected physical mechanism/stability, the vibrational SFS, non‐resonant SHS, and electrokinetic mobility measurements should be independent of the nature of the oil, with hexane and hexadecane droplets in water producing the same results. However, this is not the case. As mentioned in the introduction, there are well‐known differences in the wetting behaviour of hexane and hexadecane as they fall into non‐wetting (C_16_) or partial‐wetting (C_6_) regimes [3], with the solubility [1], contact angles and interfacial tension values being different [62, 63, 64], and MD simulations of nanoscale interfaces having very different interfacial molecular structures [65, 66]. The consequence of these differences is that hexadecane forms nanoscale oil droplets in neat water that are kinetically stable over several weeks [4, 9], but hexane does not. In contrast, hexane cannot be dispersed as stable oil droplets in water. This difference is generally explained in terms of the difference in solubility and wettability [67]. Hexadecane has a solubility of 9.3·10^−8^ mol/m^3^ (93 pM), whereas hexane has a solubility of 1.1·10^−1^ mol/m^3^ (110 mM) [1], which leads to differences in coalescence and Ostwald ripening. Freeze fracture electron microscopy experiments [1] of hexane and hexadecane droplets in water have shown that hexane droplets destabilize via coalescence while for hexadecane droplets Ostwald ripening is the main driving force. Figure 2B confirms this difference, showing the results of emulsifying neat n‐hexadecane and n‐hexane with ultrapure water using the ultrasonication procedure of Refs [4, 40, 41, 42]. Figure 2B shows the results: After taking the cuvettes out of the ultrasonic bath (∼2 mins after ending the ultrasonication), a milky dispersion is seen for n‐hexadecane in water (Figure 2Bi; typical droplet size distribution in Figure 2Biii), and a two phase‐separated system is seen for hexane in water (Figure 1B). Thus, while n‐hexadecane forms kinetically stable oil droplets in water, hexane does not. Without the possibility to create kinetically stable oil droplets in water, it is questionable if the choice of hexane is appropriate. Since coalescence is the main destabilizing process [1], it can also be expected that there are differences in surface charge, since the surface charge is the main interaction that prevents coalescence. Unfortunately, due to the unstable nature of the sample, this cannot be experimentally verified.

What can be measured, is the alkane‐water interface on nanodroplets in water stabilized with a dilute layer of negatively charged surfactants. To investigate the alkane chain surface structure on oil droplets, we performed vibrational SFS experiments of hexane and hexadecane droplets (*R* = 90 nm), that have been stabilized by a dilute layer (>4.25 nm^2^ / anion / <0.23 anions/nm^2^) of negatively charged deuterated dodecyl sulfate anions [68]. These interfaces are still abundant in oil‐water contacts and the SD^−^ surfactant anion has the same structure on both surfaces [68]. The vibrational C─H mode spectrum reflects the molecular surface conformation and differences therein and is shown in Figure 2C for hexane droplets (black data) and hexadecane droplets (blue data). Both alkane spectra are different which demonstrates the different conformation of the hexane and hexadecane molecules. Based on the different d^+^/r^+^ ratios [69], hexane has a higher relative degree of chain disorder, in agreement with MD simulations [65, 66]. The predominance of the r^−^ mode indicates that the alkyl chains are predominantly oriented more parallel to the interface than perpendicular [68]. Thus, hexane and hexadecane have different interactions with water, which result in vastly different solubilities, different interfacial structures, and very different kinetic droplet stability, rooted in, likely, different surface charging behaviour.

#### 2.3.2 Is a Cluster of a Few Hexane Molecules in Water Sufficient to Capture Electronic Polarization Effects near an Oil‐Water Interface or an Oil Droplet?

H‐bonds vary in strength as a function of local molecular structure. H‐bonds are known to display cooperative behavior, which means that the length scale over which they occur, as well as the molecular configuration matters. Specifically, the thermodynamics of hydrophobic effects in water are well known to exhibit a length‐scale crossover at approximately 1 nanometer [70]. This behaviour arises from the onset of the surface‐tension–dominated regime, which is ultimately linked to interfacial fluctuations involving both proper and improper hydrogen bonds.

Clusters of single oil molecules, an extended interface, a nanoscale droplet of oil in water or the inverse system, water‐droplets in oil, are different. Although the same physical interactions determine the behaviour of each system, collective effects can result in different macroscopic outcomes, even though the same local physical interactions are at play. Oil droplets in water, water droplets in oil and planar extended interfaces display drastically different behaviors – in terms of stability, molecular structure and electrostatics [61]. For example, oil‐in water nanodroplets are stable, while water droplets in the same oil cannot be created, even though their interfaces are composed of the same chemicals [71]. Figure 2D, replotted from Ref [61]., shows that with identical physical interactions at play on the molecular level, linearized macroscopic models generate electrostatic properties that are vastly different. The reason for this is that the collective sum of interactions over different length scales and geometries results in different eventual properties: with the same interactions between oil and water molecules, a property such as the interfacial residual electric field is ∼3 x bigger for oil droplets in water compared to a planar oil in water interface, while it nearly vanishes for water droplets in oil.

#### 2.3.3 What is the Actual Droplet Surface Charge Distribution?

The discussion about what stabilizes the droplets and what is the nature of the charge is influenced by the question of what the magnitude of the surface charge density is. This is uncertain from both the experimental and theoretical perspective. Starting from the theoretical side, studying the coupling between hydrodynamics and electronic fluctuations is currently limited by several challenges, such as finite size and affordable simulation timescales, the quality of the underlying electronic structure description, and perhaps more generally, how one quantifies local and non‐local electronic polarization at interfaces. Based on the differences between Vacha et al. [11]., Poli et al. [72]., and Zhao et al. [6] future efforts in this direction of research seem warranted until consensus is achieved to describe CT effects consistently. Another unexplored element is the connection between the Grotthuss mechanism and hydrodynamic flow.

From the experimental side, it has long been known that there are difficulties in converting measured mobilities to ζ‐potentials and ζ‐potentials to surface potentials and surface charge densities. Common assumptions are [73]: A known diffuse double layer structure with a homogeneous and uniform charge density and ion distribution, ions are represented as point charges and the only permitted sources of charge, the ζ‐potential is assumed to be equal to the surface potential, and the absence of slip. This can lead to drastic errors in the estimated surface potential. For example, in the case of silica particles in water [74], the presence of a condensed charge layer at the interface leads to much higher surface potentials (−418 mV) than those estimated from mobility recordings (−43 mV). In the case of hexadecane droplets in water discussed here, the conversion from mobility to surface potential is based on the above assumptions as well, which can lead to large errors. For example, slip is very likely present [75, 76]. Under the present experimental conditions this can lead to up to 5 x overestimations of the surface charge density. Concretely, if without slip, a mobility of −2.2 × 10^−8^ m^2^/Vs converts to a ζ‐potential of –32 mV, then with slip it converts to a –6 mV ζ‐potential. Note that the surface potential as measured by SHS is not slip dependent, here, the assumptions relate to the type of light‐matter interactions, such as the absence of dispersion [46].

Therefore, both the uncertainty in what are the best theoretical methods and what is the best interpretation to convert experimental mobility values to surface charge distributions can deviate significantly. Without convergence on both aspects, any conclusion on the absence of CT through improper H‐bonds as a stabilizing mechanism is premature.

## 3 Conclusions

In summary, we examined the molecular origin of the negative electrophoretic mobility of neat oil droplets in water. One common explanation attributes this effect to adsorption of hydroxide ions at the interface, where increasing pH would increase the number of adsorbed OH^−^ ions and thus the droplet charge and mobility. However, this intuitive picture lacks experimental support: spectroscopic probes of hydroxide (2pπ valence transition, charge‐transfer‐to‐solvent transition, and O–H^−^ stretch) and optical measurements of surface charge (second harmonic scattering) have not detected interfacial hydroxide. Moreover, hydroxide is a small, strongly hydrated ion that is expected to remain preferentially in the bulk.

An alternative mechanism invokes CT through improper H‐bonds, which also renders kinetically stable oil droplets negatively charged. In this framework, the increase in mobility with pH reflects the rise of the bulk conductivity of water: as conductivity increases, resistance to charge motion decreases and charged droplets move faster. While less intuitive, this explanation is consistent with independent molecular‐level observations, and it brings a logical explanation across a whole range of electrophoretic measurements on different materials.

Zhao et al. dismissed this mechanism based on charge‐transfer calculations for a cluster of hexane molecules in water. We consider this conclusion premature. Hexane is nine orders of magnitude more soluble in water than hexadecane and does not form kinetically stable droplets; accordingly, SFG spectra indicate different surface structures for hexane and hexadecane droplets. Moreover, simulations of a few molecules capture only local interactions, whereas collective effects across length scales can produce different macroscopic behavior, as seen for droplets, planar oil–water interfaces, and water‐in‐oil systems. Finally, although experiments agree on the sign and magnitude of electrophoretic mobility, the inferred surface charge distribution is strongly model dependent, and theoretical estimates vary widely with the chosen charge‐partitioning scheme. Reducing these uncertainties will be essential to establish the relevance of charge‐transfer effects.

## Author Contributions

**P. Singh**: writing – review and editing, visualization, formal analysis, validation, investigation, data curation. **C. E. Rani**: writing – review and editing, visualization, validation, investigation, data curation, methodology. **S. Pullanchery**: methodology, data curation, investigation, validation, supervision, writing – review and editing. **S. Roke**: writing – review and editing, writing – original draft, project administration, resources, funding acquisition, supervision, investigation, conceptualization, formal analysis, visualization.

## Conflicts of Interest

The authors declare no conflicts of interest.

## Supporting information




**Supporting File**: Experimental section for Figures 1B and 2A,B.

## Data Availability

The data that support the findings of this study are available on request from the corresponding author. The data are not publicly available due to privacy or ethical restrictions.
