# Supplementary material for: Comment on “Can Charge Transfer Across C─H···O Hydrogen Bonds Stabilize Oil Droplets in Water?”
Source: Angew Chem Int Ed Engl. 2026 May 27;65(28):e8842163. doi: 10.1002/anie.8842163 (PMC13340542; doi:10.1002/anie.8842163)
Supplement: Supplementary file 1 — Supporting File: Experimental section for Figures 1B and 2A,B. [file ANIE-65-e8842163-s001.pdf]

Supporting Information for

Comment on “Can Charge Transfer Across C─H···O Hydrogen Bonds Stabilize Oil Droplets in Water?”

P. Singh[1], C. E. Rani[1], S. Pullanchery[2], S. Roke[1]*
[1]Laboratory for fundamental BioPhotonics (LBP), Institute of Bioengineering (IBI), and Institute of Materials Science (IMX), School of Engineering (STI), and Lausanne Centre for Ultrafast Science (LACUS), École Polytechnique Fédérale de Lausanne (EPFL), CH-1015, Lausanne, Switzerland.
[2]Department of Chemistry, Texas A&M University, College Station, Texas 77843, USA.
*sylvie.roke@epfl.ch

This PDF file includes:

Materials and Methods

Materials and Methods

Chemicals

Hexadecane (highest available analytical standard, Sigma Aldrich, < 5 mL vials), D_2O (99.8% atom % D, Acros Organics), sulfuric acid (95-97%, Merck), H_2O_2 (30%, Reactolab SA), NaCl (99.999%, Acros), NaOH (99.99%, Sigma-Aldrich), and NaOD solution (40 wt.% in D_2O, 99.5 at.% D, Sigma-Aldrich), sodium thiocyanate (NaSCN, ≥ 99.99%, Sigma-Aldrich), sodium bromide (NaBr, 99.995%), sodium fluoride (NaF, 99.99%), and sodium iodide (NaI, 99.999%) were used as received. The purity of hexadecane was verified with a Zisman test[1]. The glassware used to prepare and store the nanodroplets was freshly taken out of the manufacturer's packaging and never reused after the preparation. As a first step, the glassware was cleaned with a freshly prepared piranha (3:1 H_2SO_4:H_2O_2) solution. After being immersed in the piranha solution for ~45 minutes, the glassware was rinsed copiously with ultrapure water (18.2 MΩ ·cm), obtained from a Milli-Q UF-Plus instrument (Millipore Inc.).

Reflection SFG experiment (Fig. 1B)

Reflection SFG spectroscopy was performed using an 80 W, 1030 nm Carbide laser system (Light Conversion) operating at a 10 kHz pulse repetition rate. Approximately 76 W of the output was used to pump a tunable optical parametric amplifier (OPA) system consisting of Orpheus and Lyra (Light Conversion), generating broadband infrared pulses covering 1000–4000 cm^{-1}, with a full width at half maximum (FWHM) of 200–350 cm^{-1} and pulse energies of 5–20 µJ. Approximately 10 % of the generated IR beam was directed to a custom-built FTIR spectrometer (Light Conversion) for real-time monitoring of the IR spectral profile, while the remaining portion was guided to the sample. The IR beam was routed through a periscope and focused onto the sample plane using an off-axis parabolic mirror (f = +152.4 mm, MPD169-03 Thorlabs). The polarization of the IR beam was controlled using a low-order half-wave plate (3.5 µm, WPLH05M-3500 Thorlabs).

The remaining 4 W of the fundamental output is directed into an SHBC unit (second harmonic bandwidth compressor for Carbide) to generate narrow bandwidth (FWHM 11 cm^{-1}) visible beam at 515 nm, adjusted to 7 µJ pulse energy. The visible beam is guided through a motorized delay stage to control temporal overlap. A quartz half-wave plate (85083, Edmund Optics) and Glan-Taylor polarizer (GT10-A , Thorlabs) controls the energy and polarization of the 515 nm beam. Lastly, a lens (f = +200 mm, LBF254-200A, Thorlabs) focuses the 515 nm beam on the sample plane, where it spatially and temporally overlaps with the IR beam.

The sample surface was positioned parallel to the optical table. The IR beam was incident at ~38°, while the visible beam was incident at ~53° with respect to the surface normal, giving an opening angle of 15° between the two beams. The reflected SFG signal was collected at

~50° using reflective mirrors and collimated with a lens (f = +300 mm). The SFG beam then passed through a half-wave plate (AHWP10M-580 Thorlabs) and a Glan–Taylor polarizer to control its polarization. The beam was subsequently focused onto the entrance slit of a SpectraPro HRS-300 monochromator (1800 g/mm grating) using a +50 mm lens (LBF254-050-A, Thorlabs), and detected using an iCCD camera (PiMax3).

For the measurements reported here, the IR frequency was centered at 3500 cm^{-1} with a bandwidth of 350 cm^{-1} (FWHM). SFG spectra of the $Mg(OH_2)$ crystal (BLK-MgOH2 2D Semiconductors) were recorded with an acquisition time of 120 s. Background spectra were obtained by removing the temporal overlap between the IR and visible pulses. The final SFG spectra were obtained by subtracting the background intensity and normalizing it with the IR and visible pulse energies and spectral profiles.

<u>Oil Nanodroplets in Water (Fig. 2A,B)</u>

As described previously[2], mixtures of 2 vol% hexadecane in pure H_2O or D_2O were first mixed for ~3 minutes using a vortexer (IKA® Vortex 2). These mixtures were then ultrasonicated (35 kHz, 400 W, Bandelin) until monodisperse droplets with diameters in the appropriate size range were formed and diluted as required by the different experiments. The size distributions of the nanodroplets were characterized by dynamic light scattering (DLS) using a Malvern Zetasizer Ultra instrument.

<u>Electrokinetic mobility and conductivity measurements (Fig. 2A,B)</u>

The electrophoretic mobility measurements were performed using laser Doppler velocimetry and phase analysis light scattering, employing a dynamic light scattering instrument (Malvern Zetasizer Ultra). To perform the electrophoretic mobility measurements, the nanoemulsions were diluted to 0.05 vol% by adding ultrapure water or a solution of 300 μM NaX (X = OH^-, Cl^-, Br^-, I^-, SCN^-, F^-) in ultrapure water. The conductivities of these solutions were measured using a Hanna HI5522-01 conductivity meter.

References

1. Bigelow WC, Pickett DL, Zisman WA. Oleophobic monolayers: I. Films adsorbed from solution in non-polar liquids. *J Colloid Sci* **1**, 513-538 (1946).

2. Pullanchery S, Kulik S, Okur HI, de Aguiar HB, Roke S. On the stability and necessary electrophoretic mobility of bare oil nanodroplets in water. *J Chem Phys* **152**, 241104 (2020).
